# Mitogenomic Characterization of Mining Bee Family Andrenidae (Hymenoptera: Apoidea: Anthophila) and Insights into Bee Phylogeny

**DOI:** 10.3390/biology14101374

**Published:** 2025-10-08

**Authors:** Dan Zhang

**Affiliations:** 1 School of Criminal Justice, Shandong University of Political Science and Law, Jinan 250014, China; zhangdan@sdupsl.edu.cn; 2 Characteristic Laboratory of Forensic Science in Universities of Shandong Province, Shandong University of Political Science and Law, Jinan 250014, China; 3 State Key Laboratory of Animal Biodiversity Conservation and Integrated Pest Management, Institute of Zoology, Chinese Academy of Sciences, Beijing 100101, China

**Keywords:** pollinators, Andrenidae, mitogenome, gene rearrangement, phylogeny

## Abstract

**Simple Summary:**

Bees exhibit exceptional efficiency in pollination processes. The family Andrenidae, comprising over 3000 described species, represents an important lineage of bees, yet limited molecular information has constrained insights into its evolutionary history. This study sequenced and assembled five mitochondrial genomes of Andrenidae collected from Xizang, Tibet. Comparative genomic approaches were used to analyze nucleotide composition, codon preferences, and Ka/Ks ratios of the newly obtained mitogenomes. Furthermore, previously published datasets have been integrated to reconstruct phylogenetic relationships among bees employing both Bayesian Inference and Maximum Likelihood methods. These findings significantly expand the available molecular resources for Andrenidae and offer valuable references for future investigations into the evolutionary biology of this family and bees more broadly.

**Abstract:**

Andrenidae is a major pollinator lineage with considerable ecological importance, yet limited molecular resources have impeded comprehensive understanding of its evolutionary history. This study sequenced and assembled five mitogenomes of Andrenidae, which were collected from Xizang, Tibet. Analyses included sequence size, nucleotide composition, Ka/Ks ratios, and gene rearrangements. The assembled mitogenomes ranged from 15,631 to 18,506 bp in length. AT content (%) varied between 74.46 and 79.85. Relative synonymous codon usage analysis revealed that AUU, UUA, UUU, and UUA were the most frequently preferred codons. All 13 protein-coding genes displayed Ka/Ks values below one, with *ATP8* showing the highest ratio and *COX1* the lowest. Gene rearrangements occurred in all mitogenomes, and three distinct tRNA rearrangement patterns were detected. This study provided more available molecular data for future evolutionary biology studies of Andrenidae. Additionally, 24 previously published Apoidea mitogenomes (three outgroups and 26 ingroups) were incorporated to infer phylogenetic relationships using Maximum Likelihood and Bayesian Inference methods. The results supported Melittidae as the basal lineage of bees, while Andrenidae was recovered as the sister clade to Halictidae + Colletidae.

## 1. Introduction

Bees, comprising roughly 20,900 described species [1], represent the most species-rich group of pollen-feeding insects and act as vital pollinators in ecosystems [2,3]. Bees display remarkably diverse life-history strategies, such as variable social behavior, nesting strategies, brood parasitism, and dietary specializations on specific host plants [4,5]. Those complicated traits have made bees a model group for investigating coevolution between insects and flowers, evolutionary biology, and applied ecology [2,4].

At present, bees are classified into seven families, including two long-tongued (LT) bee families, Apidae and Megachilidae, and five short-tongued (ST) bee families, Halictidae, Colletidae, Andrenidae, Stenotritidae, and Melittidae [5,6]. The validity and monophyly of each family have been continuously confirmed in subsequent studies [7,8,9]. During the early phases of research, some bee researchers, including Michener, Engel, and Alexander identified that Colletidae was the basal group of bees, and treated it as the sister group of the remaining bee families based on morphological characteristics [5,10,11,12]. With the advancement of molecular techniques, Danforth inferred the phylogenetic relationships among bee families using Maximum Likelihood (ML) and Bayesian Inference methods (BI) with multiple nuclear genes (*28S*, *EF-1α*) as well as morphological data [9]. This analysis supported Melittidae as the basal bee lineage and the sister taxon to all remaining families, and Andrenidae is the sister group of (Halictidae + (Colletidae+ Stenotritidae)). Those results have been repeatedly confirmed in subsequent research based on enrichment of ultraconserved elements (UCEs) and transcriptome [9,13,14,15]. However, the phylogenetic relationship of bees also needs to be corroborated with more phylogenetic makers.

The mining bee (Andrenidae) is a major bee family comprising over 3000 described species distributed worldwide, except in Australia [1]. Species of this group are solitary, ground-nesting bees, many of which exhibit narrow host-plant preferences supported by specialized behaviors and morphologies [5]. Andrenidae include the second largest bee genus *Andrena* (~1500 described species), mainly distributed throughout Holarctic species [4,5]. This genus is a very complicated, difficult, and interesting bee group, both for biology and taxonomy. Most andrenines are oligolectic, often specializing on plant families, such as Asteraceae, Apiaceae, Brassicaceae, Ericaceae, Fabaceae, and Rosaceae [16]. Some *Andrena* species form communal nests where males monopolize females, and studies showed that a high proportion of females mate in their natal nests before emergence, frequently with nest mates, leading to elevated inbreeding levels [17,18]. Despite existing work on this group, studies addressing molecular evolutionary mechanisms are still restricted, largely owing to inadequate sequence data. For example, among the more than 3000 species of Andrenidae, only 37 mitochondrial genomes have been published (accessed from the NCBI database on 20 Aug. 2025). This severe lack of molecular data has significantly constrained evolutionary and phylogenetic research on the group.

Mitochondrial genomes serve as widely applied molecular markers in research on insect phylogeny, evolutionary history, speciation, and phylogeography [19,20,21]. Compared with nuclear genes, mitochondrial genomes possess several advantages, including smaller molecular size, relatively simple structure, stable gene content, high copy number, and faster evolutionary rate, maternal inheritance, elevated substitution rates, and ease of sequencing making them one of the most widely used molecular markers for investigating evolutionary relationships and genetic diversity [19,22,23]. In insects, mitogenomes usually span 14–20 kb and contain 22 transfer RNAs (tRNAs), 13 protein-coding genes (PCGs), two ribosomal RNAs (rRNAs), and a non-coding control region (CR) [23]. Gene content and arrangement are generally conserved, whereas structural characteristics provide supplementary evidence for taxonomic classification [23]. The rapid growth in complete insect mitogenome datasets, driven by advances in next-generation sequencing, has greatly enhanced comparative structural analyses and improved reconstructions of evolutionary histories across lineages [22,23,24,25].

In this study, mitochondrial genomes of five Andrenidae species were sequenced, assembled, and annotated for the first time, all newly generated data were deposited in the NCBI database. Alongside one previously published mitogenome, we analyzed genome architecture, evolutionary dynamics, and substitution patterns within the group. In addition, this study combined newly sequenced data with 24 published bee mitogenomes to reconstruct the phylogenetic relationships of bees using both BI and ML approaches based on five matrices.

## 2. Materials and Methods

### 2.1. Taxon Sampling and Sequencing

This study sequenced and assembled mitogenomes of five Andrenidae species collected from Xizang, Tibet (Table 1). Specimens were preserved in 99% ethanol and then stored at −20 °C until DNA extraction and identification. All species were determined morphologically by a taxonomist (Zeqing Niu) based on morphological characters [26,27]. Genomic DNA was extracted from legs using the Qiagen DNeasy Blood & Tissue Kit (Qiagen, Venlo, The Netherlands), and DNA concentration was quantified with a Qubit^®^ 2.0 Fluorometer (ThermoFisher, USA) and the Qubit^®^ DNA Assay Kit (ThermoFisher, Waltham, MA, USA). Sequencing libraries with a 350 bp insert size were prepared and sequenced as paired-end 150 bp reads on the Illumina NovaSeq 6000 platform. Raw data were filtered with Trimmomatic v0.32 [28] to remove adapters, low-quality bases, and short reads.

### 2.2. Assembly, Annotation, and Composition Analyses

Two assembly strategies were employed for the mitogenomes. NOVOPlasty v3.8.3 (Brussels, Belgium) [29] was used with *COX1* sequences as seed and k-mer sizes of 23–39 bp. IDBA-UD v1.1.3 (Boston, MA, USA) [30] assembled Illumina reads with parameters “--mink 40 --maxk 120”. Assemblies from both pipelines were compared and merged into a consensus sequence using Geneious v2025.2.1 (Boston, MA, USA) [31]. tRNA secondary structures were predicted with tRNAscan-SE [32], while PCGs and ribosomal RNAs (rRNAs) were annotated in Geneious through alignment with related taxa. Gene boundaries of PCGs and rRNAs were validated using MEGA X [33]. SeqKit v0.16.0 (Chongqing, China) [34] was applied to examine nucleotide composition and compositional bias. The AT-skew and GC-skew were calculated as follows: AT-skew = (A − T)/(A + T), and GC-skew = (G − C)/(G + C). Relative synonymous codon usage (RSCU) values for the newly sequenced species were estimated in MEGA X. Dnasp v6 [35] was used to calculate Ka and Ks values. Circular mitogenome maps were visualized with the online tool CGview.

### 2.3. Phylogenetic Analysis

In total, we selected 29 species to infer the phylogenetic relationship of bees, containing 26 ingroups and three sphecid wasp species as outgroups based on previous studies of Apoidea [36]. Nucleotide and protein sequences were aligned using MAFFT v7.450 (Osaka, Japan) [37] with the L-INS-I strategy. Trimal v1.4.1 (Barcelona, Spain) [38] was applied with “-automated1” strategy to refine sequences. Five concatenated datasets were then generated with FASconCAT-G v1.04 (Santa Cruz, CA, USA) [39] to reconstruct phylogenetic relationships: (1) cds_fna, nucleotide sequences of all PCGs; (2) cds_faa, amino acid sequences of all PCGs; (3) cds12_fna, nucleotide sequences of PCGs excluding third codon positions; (4) cds12_rrna, nucleotide sequences of PCGs (without third codon positions) combined with two rRNAs; and (5) cds_rrna, nucleotide sequences of all PCGs and rRNAs.

ML and BI approaches were applied to reconstruct the phylogenetic relationship of bees across all datasets. For ML analyses, ModelFinder [40] in IQTREE 2 (Canberra, ACT, Australia) [41] was used to determine the best-fitting substitution for each matrix. BI trees were inferred using Phylobayes-MPI v1.9 (Montréal, QC, Canada) [42] under the site-heterogeneous mixture model (−m CAT + GTR). Two independent Markov Chain Monte Carlo (MCMC) chains were run for 10,000,000 generations each and terminated upon reaching satisfactory convergence (maxdiff < 0.3).

## 3. Results and Discussion

### 3.1. Mitogenomic Organization

**Figure 1:**
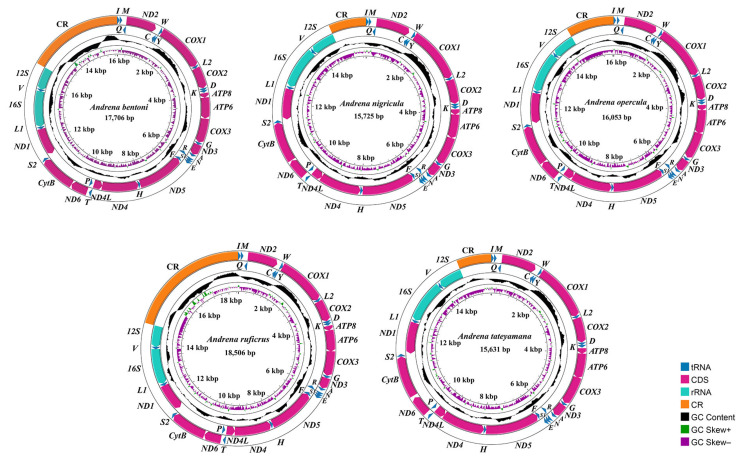
Mitogenome maps of the newly sequenced species. Arrows indicate transcription direction. Standard abbreviations denote PCGs (amaranth) and rRNAs (blue-green), and single-letters abbreviations represent tRNAs (dark blue). The thirds circle shows GC content (dark), the fourth displays GC skew (green and purple), and the innermost circle represents genome length.

Approximately six Gb of raw reads were generated for per species. Five Andrenidae species (*Andrena bentoni*, *Andrena nigricula*, *Andrena opercula*, *Andrena ruficrus*, *Andrena tateyamana*) were sequenced and assembled, all of which were complete mitogenomes, except *A. bentoni* was linear. These newly sequenced mitogenomes have been submitted to GenBank (PX147440–PX147444, Table 1). The features of the newly reported species were similar to published bee species [43,44]. For the newly sequenced species, the complete mitogenome ranged from 15,631 (*A. tateyamana*) to 18,506 bp (*A. ruficrus*), including one CR, two rRNAs, 22 tRNAs, and 13 PCGs in the structure (Table 2; Figure 1). Variation in the length of the control region contributes to interspecific differences in this region and directly influences the overall size of the mitochondrial genome [21,22]. AT skew values were positive across all species, ranging from 0.098 (*A. ruficrus*) to 0.139 (*A. bentoni*), whereas GC skew values were negative, from −0.358 (*A. opercula*) to −0.290 (*A. ruficrus*) (Table 2). AT content varied between 74.46% (*A. bentoni*) and 79.85% (*A. ruficrus*). Consistent with other insect mitogenomes [19,25], the positive AT skew and negative GC skew observed here indicate a bias toward thymine (T) and guanine (G) in Hymenoptera, in line with previous studies on bees and related taxa [45,46,47]. Gene order was conserved across all newly sequenced species.

### 3.2. Protein-Coding Genes and Evolutionary Rates

Among the newly sequenced species, the lengths of PCGs, tRNAs, and rRNAs exhibited little variation (Table 2). PCGs size ranged from 11,051 (*A. nigricula*) to 11,080 bp (*A. ruficrus*, Table 2) in length. AT skew values of newly reported mitogenomes were negative, varying from −0.060 (*A. tateyamana*) to −0.470 (*A. bentoni*), while GC skew values were negative across all mitogenomes, ranging from −0.170 (*A. bentoni*) to −0.079 (*A. ruficrus*, Table 2). The G + C content (%) ranged from 20.30 (*A. tateyamana*) to 26.68 (*A. bentoni*), and the A + T content (%) ranged from 73.30 (*A. bentoni*) to 79.70 (*A. tateyamana*, Table 2). These results indicate a strong bias toward A + T bias in nucleotide composition, consistent with patterns reported for other Hymenoptera mitogenomes [46,48]. Clary and Wolstenholme reported that such bias in nucleotide usage may result from the preferential incorporation of specific bases by DNA polymerases during mitochondrial DNA replication [49].

Among the newly obtained species, *A. tateyamana* showed the shortest CR size (937 bp), whereas *A. ruficrus* possessed the longest (3834 bp, Table 2). All PCGs started with codon ATN (Figure S1). The start codons for *ATP6*, *COX3*, and *ND4* were ATG in all species; ATA was the start codon of *ATP8*, and *CYTB* in one species; *ATP8*, *CYTB*, and *ND2* used ATT as the start codons in three species. In addition, the start codon of *ND4L* was *TTG* (Figure S1). The common stop codons were TAA or TAG, though variation existed: *ATP6* and *COX3* terminated with TA in one species, and *ND4* exhibited T as the stop codon in all newly sequenced species, which exhibited incomplete termination (Figure S2). In insect mitogenomes, PCGs frequently terminate with incomplete stop codons, which are subsequently completed through post-transcriptional polyadenylation following excision of the adjacent downstream tRNA [50,51,52].

**Figure 2:**
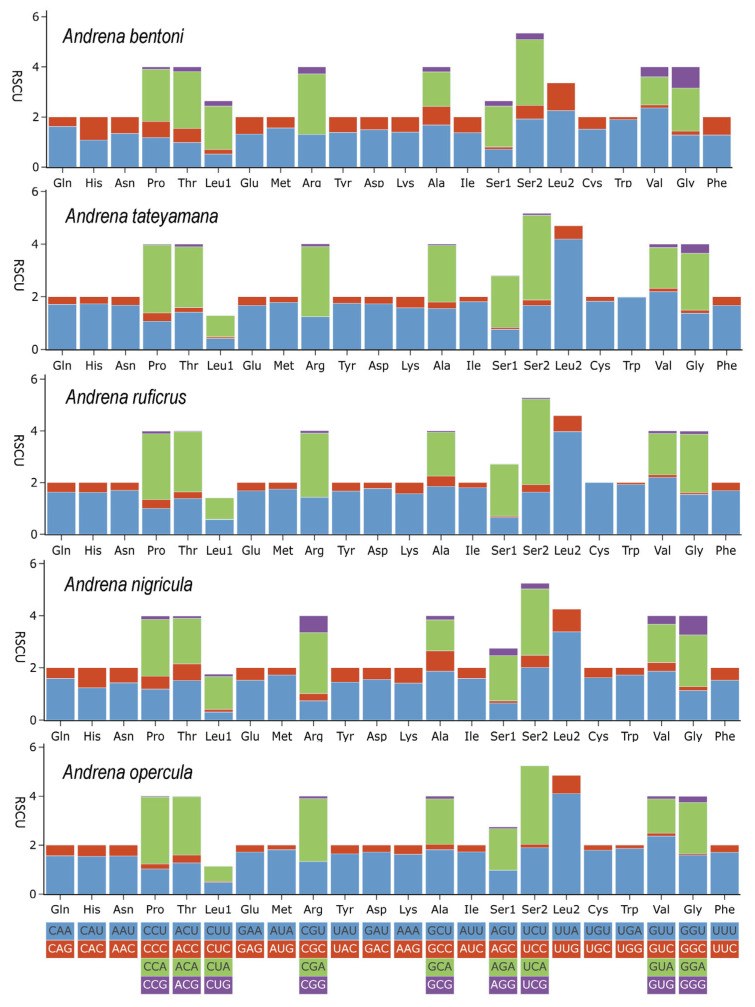
RSCU of PCGS in newly sequenced species. The X-axis represents amino acids, and the Y-axis indicates RSCU values.

The five newly sequenced mitochondrial genomes displayed consistent RSCU patterns (Figure 2), calculated from 62 codons encoding 22 amino acids in the 13 PCGs of *Andrnae* species. Preferred codons included AUU, AUA, UUA, and UUU (Tables S1–S6). Leu2, Phe, and Ile were the most frequently used amino acids, indicating a strong bias toward A/T-rich codons. For most of the newly sequenced species, codons with RSCU > 2 followed the order UUA > UCA > CGA, while *A. bentoni* showed UCA > CGA > GUU (Figure 2; Tables S1–S6). Overall, NNU and NNA were the most common codons, reflecting the strong AT bias in nucleotide composition (Tables S1–S6).

**Figure 3:**
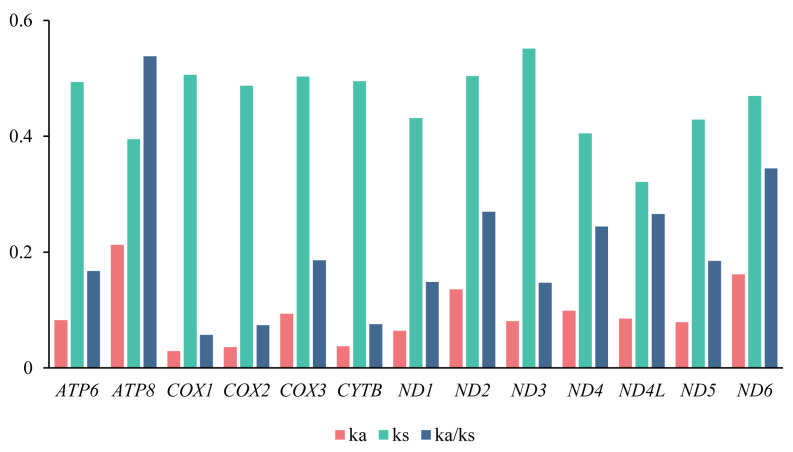
Evolutionary rates of the 13 PCGs in five newly sequenced mitogenomes. X-axis shows PCGS, and the Y-axis shows the evolutionary ratio.

The Ka/Ks ratio (ω) is widely used to evaluate the effect of natural selection on sequence evolution [53,54]. For all 13 PCGS, Ka/Ks ratios were below one, ranging from 0.058 (*COX1*) to 0.538 (*ATP8*, Figure 3), consistent with patterns reported in other insects. The relative evolutionary rates among PCGs followed the order: *ATP8* > *ND6* > *ND2* > *ND4L* > *COX3* > *ND5* > *ATP6* > *ND1* > *ND3* > *CYTB* > *COX2* > *COX1* (Figure 3). These findings indicated that most PCGs were subject to purifying selection, with deleterious mutations effectively removed during evolution (Figure 3). However, the strength of purifying selection varied across different genes. Specifically, *ATP8*, *ND6*, and *ND2* displayed relatively higher ω values, suggesting that they were subjected to more relaxed purifying selection, potentially reflecting functional constraints that were less stringent compared to other PCGs, which were pivotal in cellular energy production. In contrast, *COX2* and *COX1* showed low ω values, indicating that they were under strong purifying selection and have remained relatively conserved during evolution. These patterns were consistent with previous studies on bees, further supporting the notion that genes associated with the oxidative phosphorylation pathway, particularly cytochrome oxidase subunits, were typically constrained by evolutionary pressures due to their indispensable role in cellular respiration and energy metabolism [55,56].

All 22 tRNAs were identified in all newly sequenced species, with lengths ranging from 57 to 71 bp. The tRNAs exhibited the following features: AT content varied from 81.32% (*A. nigricula*) to 83.93% (*A. opercula*); GC content ranged from 16.07% (*A. opercula*) to 18.61% (*A. nigricula*); and both AT and GC skews were positive (Table 2).

The large subunit rRNA (*rrnL*) and the small subunit rRNA (*rrnS*) were detected in the newly obtained mitogenome. *rrnL* length ranged from 1267 (*A. ruficrus*) to 1280 bp (*A. nigricula*), and *rrnS* ranged from 743 (*A. tateyamana*) to 776 bp (*A. nigricula*). AT content (%) of *rrnL* ranged from 79.69 (*A. nigricula*) to 82.19 (*A. tateyamana*), while for *rrnS* ranged from 77.12 (*A. bentoni*) to 81.70 (*A. tateyamana*). The AT skew of *rrnL* (−0.120 to −0.078) and *rrnS* was negative (−0.117 to −0.028), while GC skew was positive (Table 2).

### 3.3. Gene Rearrangement

Although relatively uncommon, mitochondrial gene rearrangements have been documented in Hymenoptera, Thysanoptera, Trichoptera, Hemiptera, and Psocodea [20,47,57,58,59]. Four principal types are currently recognized: local inversion, remote inversion, gene shuffling, and translocation [48]. For Apoidea, inversion and inverse transposition occurred relatively frequently, paralleling patterns observed in other hymenopterans [47,48,60]. Furthermore, four main hypotheses have been proposed regarding the mechanisms underlying mitochondrial gene rearrangements in animals: Tandem duplication/random loss, TDRL [61], Tandem duplication/nonrandom loss, TDNL [62], recombination [63], and Anticodon mutation [64]. The tandem duplication/random loss (TDRL) and recombination hypotheses have been widely applied to explain mitochondrial rearrangements in insects. The former is often invoked to account for gene translocations and gene shuffling events, whereas the latter is primarily used to explain inversions and transpositions [48]. In contrast, the replication/anticodon mutation and tandem duplication/non-random loss hypotheses are relatively uncommon in insects and even across arthropods. More broadly, members of Hymenoptera exhibited particularly extensive mitochondrial rearrangements, which often provide valuable phylogenetic information [19,45,48,57]. Despite this, the processes driving such rearrangements in Andrenidae remained poorly understood, largely due to the scarcity of available mitogenome data.

**Figure 4:**
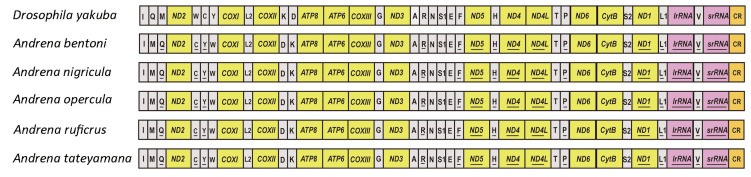
Gene order of newly sequenced mitogenomes. PCGs, rRNAs, tRNAs, and the control region are marked with yellow, pink, grey and orange.

The gene order of *Drosophila yakuba* was chosen as the ancestral reference [49]. In contrast to the putative ancestral insect mitogenome, all five newly sequenced species exhibited gene rearrangement. Totally, three gene rearrangement patterns were found, and all rearranged genes were tRNA (Figure 4). The gene order of all newly sequenced species was the same. The gene cluster *trnI-trnQ-trnM* was rearranged to *trnM-trnI-trnQ* (Figure 4); the *trnK-trnD* tRNA block was rearranged: *COX2-trnK-trnD* to *COX2-trnD-trnK*, and the *trnW-trnC-trnY* was inverted to *trnC-trnY-trnW*, which were consistent with previously published mitogenomes of the genus Andrena [44].

Gene shuffling events, including *trnI-trnQ-trnM*, *trnW-trnC-trnY*, and *trnK-trnD*, represent recurring rearrangement patterns in bees and have been widely regarded as a hallmark of Hymenoptera mitochondrial evolution [43,47,48,60]. The segment *CR-trnI-trnQ-trnM-nad2-trnW-trnC-trnY* has been identified as the principal rearrangement hotspot in bees, and our results confirm that Andrenidae also exhibits frequent changes in this region (Figure 4). The persistence of rearrangements within this cluster likely reflects structural susceptibility of the control region-proximal tRNAs, which may facilitate events such as slipped-strand mispairing or tandem duplication followed by random loss [64,65]. The recurrent emergence of these rearrangements across Apoidea underscore their phylogenetic informativeness, offering not only a robust marker for resolving hymenopteran relationships but also key insights into the molecular mechanisms driving mitogenome plasticity in insects [19,25,44]. The mitochondrial genome theoretically contains 37 genes with substantial potential for rearrangement, making it unlikely for distinct species to retain identical gene orders. Therefore, conserved sequences more likely reflect shared evolutionary origins [19,45,57].

In this study, all rearrangement patterns were consistently observed across all newly sequenced species, suggesting that tRNA gene rearrangements may represent a characteristic feature of Andrenidae. By contrast, many other Hymenoptera and insect lineages display more variable mitochondrial gene arrangements [44,45]. Compared the gene order of bee families, we found that Halictidae exhibited diverse and complex rearrangement patterns [43,45], highlighting that gene rearrangement may include phylogenetic markers for elucidating evolutionary relationships within the family. Unfortunately, due to sample limitations, our understanding of the evolution underlying gene rearrangement in bees remains unclear.

### 3.4. Phylogenetic Relationships

**Figure 5:**
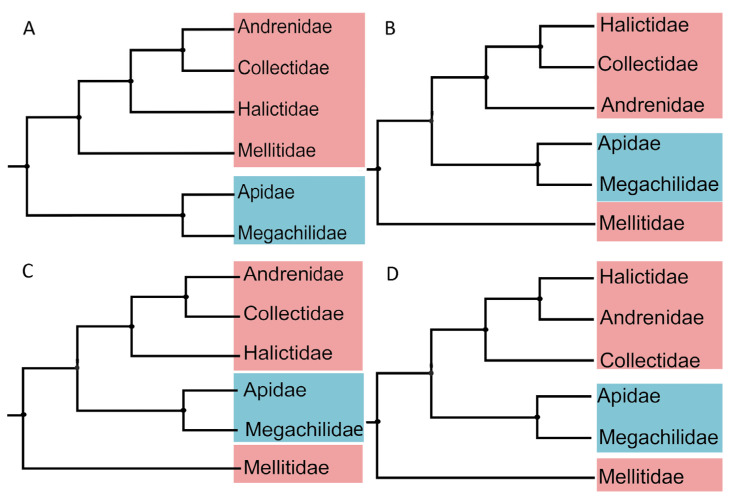
Phylogenetic relationship of bee families from different matrices. (**A**), topology 1; (**B**), topology2; (**C**), topology 3; (**D**), topology 4. LT-bees and ST-bees were marked with blue, and pink, respectively.

**Figure 6:**
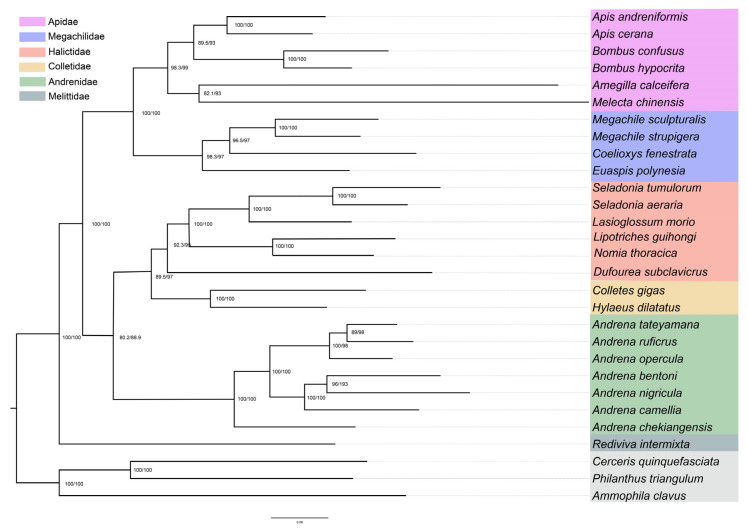
Phylogenetic relationship of bees based on the analysis of cds_fna with the partition model in IQTREE. Support values on nodes indicate SH-aLRT/UFBoot2.

In this study, we used 26 bee species and three outgroups to infer the phylogenetic relationships of bees. Five concatenated datasets were analyzed using BI and ML approaches: cds_faa (3264 sites), cds_fna (9792 sites), cds_rrna (11,736 sites), cds12_fna (6528 sites), and cds12_rrna (8472 sites), and four topologies were generated finally (Figure 5, Figure 6 and Figures S3–S11). All of them supported the monophyly of LT bees (Figure 5, Figure 6 and Figures S3–S11). Topology 1 (T1) was inferred from cds_faa using BI and ML methods (Figure 5A, Figures S3 and S4). This topology suggested that ST bees were monophyletic group. Topology 2 (T2) was generated by matrices cds_fna using BI and ML model, and cds12_fna using BI methods (Figure 5B, Figure 6, Figures S5 and S6). This topology suggested that Mellitidae was the basal group of bees, and Andrenidea was the sister group of Halictidae + Colletidae. Topology 3 (T3) was generated using cds12_rrna based on ML and BI methods (Figure 5C, Figures S7 and S8). This topology supported that Mellitidae was the basal lineage of bees, and Halictidae was the sister group of Andrenidae + Colletidae. Topology 4 (T4) was generated using cds_rrna based on ML and BI methods, and cds12_fna using ML methods (Figure 5D, Figures S9–S11). This topology supported that Mellitidae was the basal group of bees, and Colletidae was the sister group of Halictidae + Andrenidae.

Our findings strongly supported the monophyly of all bee families, except Melittidae, for which only a single species was included. All topologies, except T1, placed Melittidae as the basal lineage of bees (Figure 5), which has been confirmed by several previous studies with morphological and molecular data [9,44,66]. But, Kahnt et al. used two rRNAs and 13 PCGs to infer the phylogenetic relationship of bees with BI and ML methods, finding that Melittidae was the sister group of Colletidae in 2015 [67]. However, their analysis did not include Halictidae and Andrenidae, and most bee researchers disagree with this point [14,15].

The monophyly of LT bees was robustly supported, consistent with evidence from both morphological and molecular studies [4,9]. Considering Melittidae as the sister group to the remaining bee families, ST bees were inferred to be paraphyletic. Within ST bees, the relationship of (Andrenidae + (Halictidae + Colletidae)) was robustly recovered using cds_fna and cds12_fna (Figure 6, Figures S5 and S6), consistent with the currently accepted results [4,8,36,68]. This suggests that a total-evidence strategy based on nucleotide sequences may provide an effective approach for analyzing mitochondrial genome data [69].

However, the phylogenetic positions of Halictidae, Colletidae, and Andrenidae were highly unstable across different datasets in this study (Figure 5), likely reflecting conflicting signals among mitochondrial genes. Many studies have reported that incongruent phylogenetic signals are frequently observed between nuclear and mitochondrial genes [19,25,46,68]. When mitochondrial genomes were applied in phylogenetic analyses, variation in gene choice, site selection, or evolutionary models could yield different results [68,70,71]. In addition, the high AT content and compositional heterogeneity of insect mitochondrial genomes might also introduce systematic errors in phylogenetic analyses, leading to inconsistencies with mitochondrial and nuclear gene data [72,73]. Similarity in nucleotide composition led to the incorrect inference of distantly related taxa as closely related [70,71]. Such biases have been reported in mitochondrial phylogenomic studies of Coleoptera, Hymenoptera, and Hemiptera [70,74,75]. Variable topologies among different matrices and methods (BI, ML) indicated that further phylogenomic and taxonomic research. Future work should aim to integrate mitochondrial and nuclear datasets, apply site-heterogeneous models, and incorporate additional phylogenetic markers such as ultraconserved elements (UCEs) or single-copy orthologs (USCOs). USCOs have already been successfully applied in phylogenetic analyses of other insect groups, demonstrating their utility for resolving deep divergences and improving tree robustness. Such integrative approaches are expected to enhance phylogenetic resolution and support for deep divergences within *Andrenidae* and across bees, providing a more comprehensive understanding of their evolutionary history [76,77,78].

## 4. Conclusions

Herein, five mitogenomes of Andrenidae were newly sequenced, assembled, and annotated. Comparative analyses found that all newly obtained mitogenomes shared similar structural characters and nucleotide compositions. Gene rearrangements were exhibited in each of the newly reported mitogenomes. These new genomic resources not only enrich the limited mitochondrial dataset available for this underrepresented bee family but also provide valuable insights into gene rearrangements, codon usage patterns, and phylogenetic relationship. By incorporating 24 previously published species, we reconstructed the phylogenetic relationship of bees using BI and ML approaches. The results showed that Melittidae was the basal group of bees, and Andrenidae was the sister group of Halictidae + Colletidae. Further studies, incorporating additional samples and employing more phylogenetic markers, such as UCEs and USCOs are needed to resolve the phylogenetic relationships within the family and to advance our understanding of the evolutionary biology of bees.

## Figures and Tables

**Table 1:** Sampling information for the species newly sequenced in this study.

Species	Location	Longitude and Latitude	Elevation (m)	Date	Collector	Accession Number
*Andrena bentoni*	Xizang, Yadong	E88.9330, N27.5198	3660	2023.7.9	Qingtao Wu	PX147440
*Andrena nigricula*	Xizang, Jilong	E85.2393, N28.5808	4082	2023.7.15	Qingtao Wu	PX147441
*Andrena opercula*	Xizang, Jilong	E85.3086, N28.3987	2858	2023.7.17	Qingtao Wu	PX147442
*Andrena ruficrus*	Xizang, Jilong	E85.4555, N28.3881	3265	2023.7.22	Qingtao Wu	PX147443
*Andrena tateyamana*	Xizang, Bomi	E94.8044, N30.2510	2262	2023.6.26	Qingtao Wu	PX147444

**Table 2:** Base composition of newly sequenced species.

Samples	Regions	Length (bp)	A (%)	T (%)	C (%)	G (%)	A + T (%)	G + C (%)	AT-Skew	GC-Skew
*Andrena bentoni*	Whole genome	17,706	42.49	32.11	17.19	8.14	74.60	25.32	0.139	−0.357
	PCGs	11,074	34.92	38.38	15.61	11.07	73.30	26.68	−0.047	−0.170
	Site 1	3692	40.52	31.29	13.89	14.30	71.81	28.19	0.128	0.014
	Site 2	3691	23.09	49.75	16.36	10.80	72.84	27.16	−0.366	−0.205
	Site 12	7383	31.80	40.52	15.13	12.55	72.33	27.67	−0.121	−0.093
	Site 3	3691	41.15	34.10	16.57	8.10	75.25	24.68	0.094	−0.343
	tRNA	1429	42.20	39.19	7.56	11.06	81.39	18.61	0.037	0.188
	l-rRNA	1278	35.99	43.74	6.49	13.77	79.73	20.27	−0.097	0.359
	s-rRNA	765	35.42	41.70	6.14	16.73	77.12	22.88	−0.081	0.463
	CR	2975	40.40	35.93	13.78	9.55	76.33	23.33	0.059	−0.181
*Andrena nigricula*	Whole genome	15,725	42.52	34.10	15.80	7.55	76.62	23.35	0.110	−0.353
	PCGs	11,051	36.34	40.00	13.43	10.23	76.34	23.66	−0.048	−0.135
	Site 1	3685	43.63	32.06	11.62	12.69	75.69	24.31	0.153	0.044
	Site 2	3684	23.09	50.26	15.90	10.76	73.34	26.66	−0.370	−0.193
	Site 12	7369	33.36	41.16	13.76	11.72	74.52	25.48	−0.105	−0.080
	Site 3	3682	42.30	37.69	12.76	7.25	79.99	20.01	0.058	−0.275
	tRNA	1424	40.66	40.66	8.01	10.67	81.32	18.68	0.000	0.142
	l-rRNA	1280	36.72	42.97	5.94	14.37	79.69	20.31	−0.078	0.415
	s-rRNA	776	39.56	41.88	5.15	13.40	81.44	18.56	−0.028	0.445
	CR	1032	42.73	32.75	18.31	5.72	75.48	24.03	0.132	−0.524
*Andrena opercula*	Whole genome	16,053	45.21	34.57	13.68	6.47	79.78	20.15	0.133	−0.358
	PCGs	11,071	37.68	42.02	11.04	9.26	79.69	20.31	−0.054	−0.088
	Site 1	3691	43.53	34.17	9.72	12.59	77.69	22.31	0.120	0.129
	Site 2	3690	23.43	50.34	15.14	11.09	73.77	26.23	−0.365	−0.155
	Site 12	7381	33.48	42.25	12.43	11.84	75.73	24.27	−0.116	−0.024
	Site 3	3690	46.07	41.54	8.27	4.11	87.61	12.39	0.052	−0.336
	tRNA	1419	43.55	40.38	6.77	9.30	83.93	16.07	0.038	0.157
	l-rRNA	1268	36.51	45.58	5.52	12.38	82.10	17.90	−0.110	0.383
	s-rRNA	746	37.40	43.83	5.63	13.14	81.23	18.77	−0.079	0.400
	CR	1357	49.01	29.26	18.13	2.73	78.27	20.86	0.252	−0.738
*Andrena ruficrus*	Whole genome	18,506	43.82	36.02	12.98	7.14	79.84	20.13	0.098	−0.290
	PCGs	11,080	37.60	42.08	10.96	9.36	79.68	20.32	−0.056	−0.079
	Site 1	3694	42.88	33.18	10.76	13.18	76.06	23.94	0.128	0.101
	Site 2	3693	23.35	50.67	15.07	10.91	74.02	25.98	−0.369	−0.160
	Site 12	7387	33.12	41.92	12.92	12.04	75.04	24.96	−0.117	−0.035
	Site 3	3693	46.56	42.40	7.04	4.00	88.96	11.04	0.047	−0.276
	tRNA	1423	43.08	40.34	6.89	9.70	83.42	16.58	0.033	0.169
	l-rRNA	1267	35.44	45.15	6.08	13.34	80.58	19.42	−0.120	0.374
	s-rRNA	744	36.83	43.55	6.05	13.58	80.38	19.62	−0.084	0.384
	CR	3834	42.72	33.72	16.33	5.55	76.44	21.88	0.118	−0.493
*Andrena tateyamana*	Whole genome	15,631	44.17	35.68	13.27	6.83	79.85	20.11	0.106	−0.320
	PCGs	11,059	37.47	42.23	11.05	9.25	79.70	20.30	−0.060	−0.089
	Site 1	3687	42.27	34.03	10.95	12.74	76.30	23.70	0.108	0.075
	Site 2	3686	23.08	50.65	15.07	11.20	73.74	26.26	−0.374	−0.147
	Site 12	7373	32.68	42.34	13.01	11.97	75.02	24.98	−0.129	−0.042
	Site 3	3686	47.04	42.02	7.13	3.81	89.06	10.94	0.056	−0.303
	tRNA	1431	43.26	39.97	7.27	9.50	83.23	16.77	0.040	0.133
	l-rRNA	1269	37.27	44.92	5.67	12.14	82.19	17.81	−0.093	0.363
	s-rRNA	743	37.28	44.41	4.98	13.32	81.70	18.30	−0.087	0.456
	CR	937	43.65	37.79	11.95	7.38	81.44	19.33	0.072	−0.236

## Data Availability

The data are contained within the article or Supplementary Materials.

## Supplementary Materials

The following supporting information can be downloaded at: https://www.mdpi.com/article/10.3390/biology14101374/s1. Table S1: RSCUs of *Andrena bentoni*. Table S2: RSCUs *Andrena nigricula*. Table S3: RSCUs of *Andrena opercula*. Table S4: RSCUs of *Andrena ruficrus*. Table S5: RSCUs of *Andrena tateyamana*. Figure S1. Start codons of PCGs for newly obtained mitogenomes. *X*-axis shows PCGS, and the *Y*-axis indicates the number of species. Figure S2. Stop codons of PCGs among newly reported mitogenomes. *X*-axis shows PCGS, and the *Y*-axis indicates the number of species. Figure S3: ML analysis of bees based on cds_faa using partitioned model in IQTREE. Nodes support values indicate SH-aLRT/UFBoot2. Figure S4. BI tree of bees based on cd_faa with a GTR + CAT model in phylobayes. Nodes support values indicate Bayesian posterior probabilities. Figure S5: ML analysis of bees based on cds_rrna using partitioned model in IQTREE. Nodes support values indicate SH-aLRT/UFBoot2. Figure S6: BI tree of bees based on cd_rrna with a GTR + CAT model in phylobayes. Nodes support values indicate Bayesian posterior probabilities. Figure S7: BI tree of bees based on cd_fna with a GTR + CAT model in phylobayes. Nodes support values indicate Bayesian posterior probabilities. Figure S8. BI tree of bees based on cd12_fna with a GTR + CAT model in phylobayes. Nodes support values indicate Bayesian posterior probabilities. Figure S9. ML analysis of bees based on cds12_fna using partitioned model in IQTREE. Nodes support values indicate SH-aLRT/UFBoot2. Figure S10. ML analysis of bees based on cds12_rrna using partitioned model in IQTREE. Nodes support values indicate SH-aLRT/UFBoot2. Figure S11. BI tree of bees based on cds_rrna with a GTR + CAT model in phylobayes. Nodes support values indicate Bayesian posterior probabilities.
