# Supplementary material for: Mitogenomic Characterization of Mining Bee Family Andrenidae (Hymenoptera: Apoidea: Anthophila) and Insights into Bee Phylogeny
Source: Biology (Basel). 2025 Oct 8;14(10):1374. doi: 10.3390/biology14101374 (PMC12561233; doi:10.3390/biology14101374)
Supplement: Supplementary file 1 [file biology-14-01374-s001.zip › supplementary.pdf]

Table S1. RSCUs of *Andrena bentoni*

Codon	Count	RSCU	Codon	Count	RSCU
UUU(F)	224	1.28	UCU(S)	81	1.92
UUC(F)	126	0.72	UCC(S)	23	0.54
UUA(L)	186	2.25	UCA(S)	111	2.63
UUG(L)	92	1.11	UCG(S)	11	0.26
CUU(L)	43	0.52	CCU(P)	35	1.18
CUC(L)	15	0.18	CCC(P)	19	0.64
CUA(L)	143	1.73	CCA(P)	62	2.08
CUG(L)	17	0.21	CCG(P)	3	0.1
AUU(I)	283	1.37	ACU(T)	42	0.98
AUC(I)	130	0.63	ACC(T)	24	0.56
AUA(M)	275	1.56	ACA(T)	97	2.27
AUG(M)	77	0.44	ACG(T)	8	0.19
GUU(V)	118	2.36	GCU(A)	34	1.68
GUC(V)	6	0.12	GCC(A)	15	0.74
GUA(V)	56	1.12	GCA(A)	28	1.38
GUG(V)	20	0.4	GCG(A)	4	0.2
UAU(Y)	155	1.38	UGU(C)	28	1.51
UAC(Y)	70	0.62	UGC(C)	9	0.49
UAA(*)	12	2	UGA(W)	80	1.9
UAG(*)	0	0	UGG(W)	4	0.1
CAU(H)	34	1.08	CGU(R)	14	1.3
CAC(H)	29	0.92	CGC(R)	0	0
CAA(Q)	39	1.62	CGA(R)	26	2.42
CAG(Q)	9	0.38	CGG(R)	3	0.28
AAU(N)	143	1.34	AGU(S)	30	0.71
AAC(N)	70	0.66	AGC(S)	4	0.09
AAA(K)	93	1.39	AGA(S)	69	1.63
AAG(K)	41	0.61	AGG(S)	9	0.21
GAU(D)	53	1.49	GGU(G)	53	1.28
GAC(D)	18	0.51	GGC(G)	6	0.15
GAA(E)	48	1.32	GGA(G)	71	1.72
GAG(E)	25	0.68	GGG(G)	35	0.85

* Stop codon

Table S2. RSCUs *Andrena nigricula*

Codon	Count	RSCU	Codon	Count	RSCU
UUU(F)	259	1.52	UCU(S)	86	2.01
UUC(F)	82	0.48	UCC(S)	20	0.47
UUA(L)	282	3.38	UCA(S)	109	2.55
UUG(L)	73	0.87	UCG(S)	9	0.21
CUU(L)	26	0.31	CCU(P)	35	1.18
CUC(L)	8	0.1	CCC(P)	15	0.5
CUA(L)	104	1.25	CCA(P)	65	2.18
CUG(L)	8	0.1	CCG(P)	4	0.13
AUU(I)	357	1.59	ACU(T)	64	1.51
AUC(I)	91	0.41	ACC(T)	27	0.64
AUA(M)	313	1.72	ACA(T)	74	1.75
AUG(M)	52	0.28	ACG(T)	4	0.09
GUU(V)	80	1.87	GCU(A)	36	1.87
GUC(V)	14	0.33	GCC(A)	15	0.78
GUA(V)	63	1.47	GCA(A)	23	1.19
GUG(V)	14	0.33	GCG(A)	3	0.16
UAU(Y)	160	1.45	UGU(C)	26	1.62
UAC(Y)	60	0.55	UGC(C)	6	0.38
UAA(*)	8	1.6	UGA(W)	74	1.72
UAG(*)	2	0.4	UGG(W)	12	0.28
CAU(H)	40	1.23	CGU(R)	8	0.74
CAC(H)	25	0.77	CGC(R)	3	0.28
CAA(Q)	35	1.59	CGA(R)	25	2.33
CAG(Q)	9	0.41	CGG(R)	7	0.65
AAU(N)	153	1.42	AGU(S)	28	0.65
AAC(N)	63	0.58	AGC(S)	4	0.09
AAA(K)	98	1.41	AGA(S)	74	1.73
AAG(K)	41	0.59	AGG(S)	12	0.28
GAU(D)	48	1.55	GGU(G)	44	1.12
GAC(D)	14	0.45	GGC(G)	6	0.15
GAA(E)	57	1.52	GGA(G)	78	1.99
GAG(E)	18	0.48	GGG(G)	29	0.74

* Stop codon

Table S3. RSCUs of *Andrena opercula*

**Codon**	**Count**	**RSCU**	**Codon**	**Count**	**RSCU**
UUU(F)	310	1.7	UCU(S)	80	1.89
UUC(F)	55	0.3	UCC(S)	6	0.14
UUA(L)	328	4.1	UCA(S)	135	3.2
UUG(L)	60	0.75	UCG(S)	1	0.02
CUU(L)	38	0.47	CCU(P)	29	1.02
CUC(L)	3	0.04	CCC(P)	6	0.21
CUA(L)	50	0.62	CCA(P)	78	2.74
CUG(L)	1	0.01	CCG(P)	1	0.04
AUU(I)	417	1.72	ACU(T)	51	1.27
AUC(I)	68	0.28	ACC(T)	13	0.32
AUA(M)	326	1.81	ACA(T)	96	2.39
AUG(M)	35	0.19	ACG(T)	1	0.02
GUU(V)	86	2.37	GCU(A)	33	1.81
GUC(V)	4	0.11	GCC(A)	4	0.22
GUA(V)	51	1.41	GCA(A)	34	1.86
GUG(V)	4	0.11	GCG(A)	2	0.11
UAU(Y)	180	1.64	UGU(C)	34	1.79
UAC(Y)	40	0.36	UGC(C)	4	0.21
UAA(*)	11	1.83	UGA(W)	80	1.86
UAG(*)	1	0.17	UGG(W)	6	0.14
CAU(H)	47	1.54	CGU(R)	14	1.33
CAC(H)	14	0.46	CGC(R)	0	0
CAA(Q)	39	1.56	CGA(R)	27	2.57
CAG(Q)	11	0.44	CGG(R)	1	0.1
AAU(N)	168	1.55	AGU(S)	41	0.97
AAC(N)	49	0.45	AGC(S)	0	0
AAA(K)	114	1.62	AGA(S)	72	1.7
AAG(K)	27	0.38	AGG(S)	3	0.07
GAU(D)	58	1.71	GGU(G)	63	1.59
GAC(D)	10	0.29	GGC(G)	2	0.05
GAA(E)	64	1.71	GGA(G)	83	2.1
GAG(E)	11	0.29	GGG(G)	10	0.25

* Stop codon

Table S4. RSCUs of *Andrena ruficrus*

Codon	Count	RSCU	Codon	Count	RSCU
UUU(F)	300	1.69	UCU(S)	68	1.63
UUC(F)	56	0.31	UCC(S)	12	0.29
UUA(L)	328	3.97	UCA(S)	138	3.31
UUG(L)	51	0.62	UCG(S)	2	0.05
CUU(L)	47	0.57	CCU(P)	29	1
CUC(L)	1	0.01	CCC(P)	10	0.34
CUA(L)	69	0.83	CCA(P)	74	2.55
CUG(L)	0	0	CCG(P)	3	0.1
AUU(I)	421	1.8	ACU(T)	52	1.38
AUC(I)	48	0.2	ACC(T)	10	0.26
AUA(M)	319	1.75	ACA(T)	88	2.33
AUG(M)	46	0.25	ACG(T)	1	0.03
GUU(V)	88	2.2	GCU(A)	37	1.85
GUC(V)	4	0.1	GCC(A)	8	0.4
GUA(V)	64	1.6	GCA(A)	34	1.7
GUG(V)	4	0.1	GCG(A)	1	0.05
UAU(Y)	184	1.67	UGU(C)	35	2
UAC(Y)	36	0.33	UGC(C)	0	0
UAA(*)	11	1.83	UGA(W)	83	1.93
UAG(*)	1	0.17	UGG(W)	3	0.07
CAU(H)	52	1.62	CGU(R)	15	1.43
CAC(H)	12	0.38	CGC(R)	0	0
CAA(Q)	40	1.63	CGA(R)	26	2.48
CAG(Q)	9	0.37	CGG(R)	1	0.1
AAU(N)	189	1.7	AGU(S)	27	0.65
AAC(N)	33	0.3	AGC(S)	2	0.05
AAA(K)	108	1.57	AGA(S)	84	2.01
AAG(K)	30	0.43	AGG(S)	1	0.02
GAU(D)	55	1.77	GGU(G)	62	1.54
GAC(D)	7	0.23	GGC(G)	3	0.07
GAA(E)	63	1.68	GGA(G)	91	2.26
GAG(E)	12	0.32	GGG(G)	5	0.12

* Stop codon

Table S5. RSCUs of *Andrena tateyamana*

Codon	Count	RSCU	Codon	Count	RSCU
UUU(F)	305	1.67	UCU(S)	70	1.67
UUC(F)	60	0.33	UCC(S)	9	0.21
UUA(L)	345	4.19	UCA(S)	135	3.22
UUG(L)	42	0.51	UCG(S)	3	0.07
CUU(L)	34	0.41	CCU(P)	31	1.07
CUC(L)	5	0.06	CCC(P)	9	0.31
CUA(L)	67	0.81	CCA(P)	75	2.59
CUG(L)	1	0.01	CCG(P)	1	0.03
AUU(I)	422	1.81	ACU(T)	55	1.41
AUC(I)	45	0.19	ACC(T)	7	0.18
AUA(M)	316	1.78	ACA(T)	90	2.31
AUG(M)	39	0.22	ACG(T)	4	0.1
GUU(V)	86	2.19	GCU(A)	31	1.55
GUC(V)	5	0.13	GCC(A)	5	0.25
GUA(V)	61	1.55	GCA(A)	43	2.15
GUG(V)	5	0.13	GCG(A)	1	0.05
UAU(Y)	192	1.75	UGU(C)	32	1.83
UAC(Y)	27	0.25	UGC(C)	3	0.17
UAA(*)	11	1.83	UGA(W)	86	1.98
UAG(*)	1	0.17	UGG(W)	1	0.02
CAU(H)	57	1.73	CGU(R)	13	1.24
CAC(H)	9	0.27	CGC(R)	0	0
CAA(Q)	42	1.71	CGA(R)	28	2.67
CAG(Q)	7	0.29	CGG(R)	1	0.1
AAU(N)	188	1.68	AGU(S)	32	0.76
AAC(N)	36	0.32	AGC(S)	3	0.07
AAA(K)	106	1.58	AGA(S)	82	1.96
AAG(K)	28	0.42	AGG(S)	1	0.02
GAU(D)	51	1.73	GGU(G)	55	1.37
GAC(D)	8	0.27	GGC(G)	5	0.12
GAA(E)	61	1.67	GGA(G)	87	2.16
GAG(E)	12	0.33	GGG(G)	14	0.35

* Stop codon

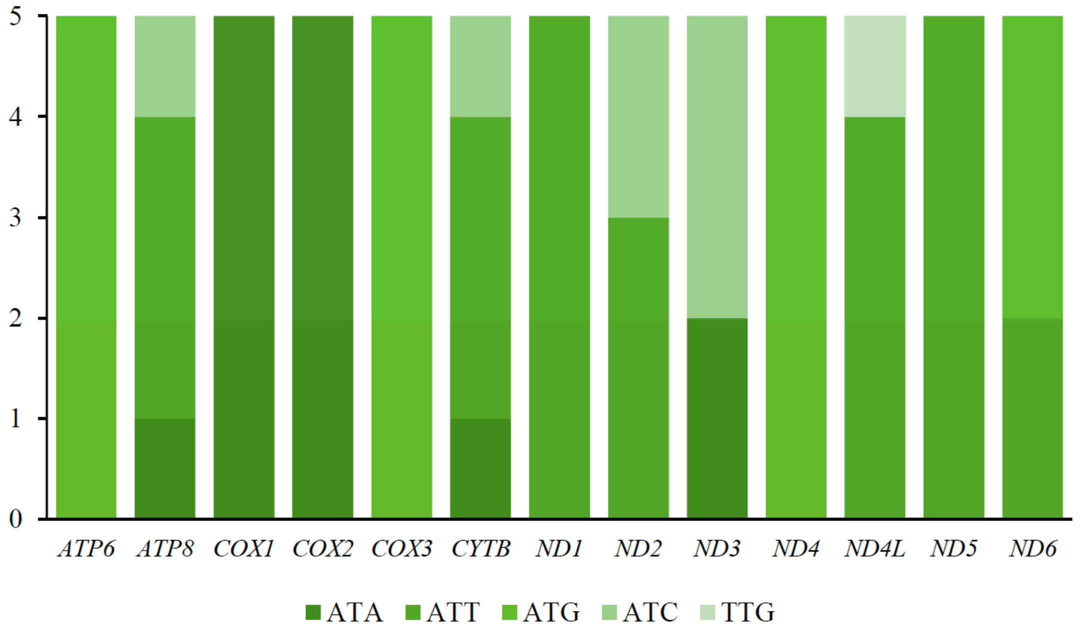

Figure S1. Start codons of PCGs for newly obtained mitogenomes. X-axis shows PCGS, and the Y-axis indicates number of species.

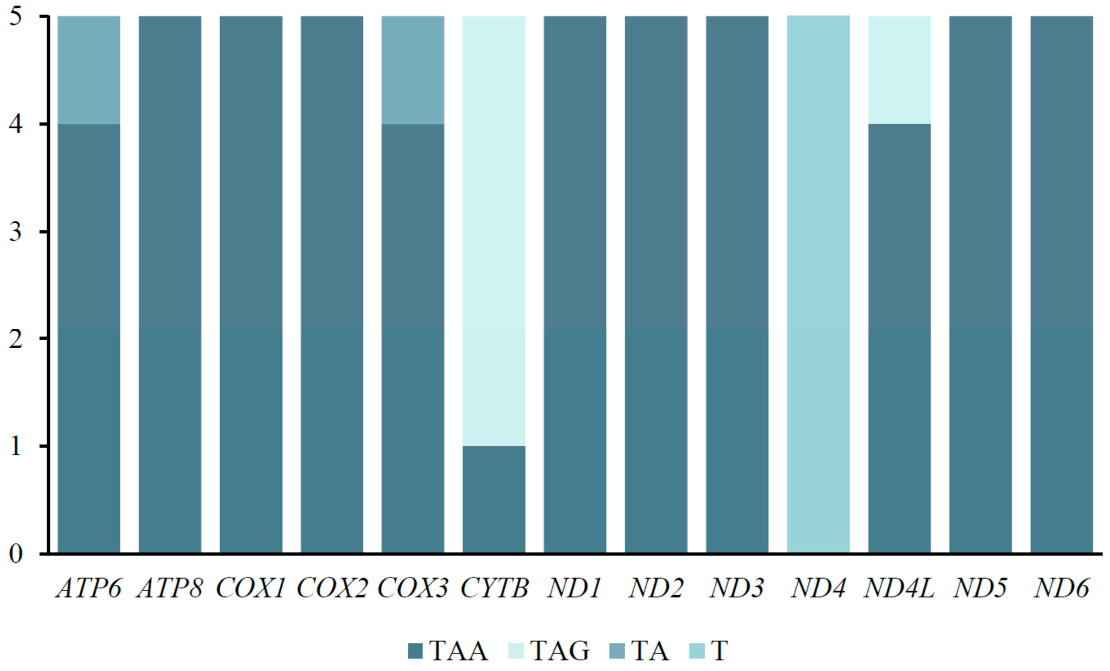

Figure S2. Stop codons of PCGs among newly reported mitogenomes. X-axis shows PCGS, and the Y-axis indicates number of species.

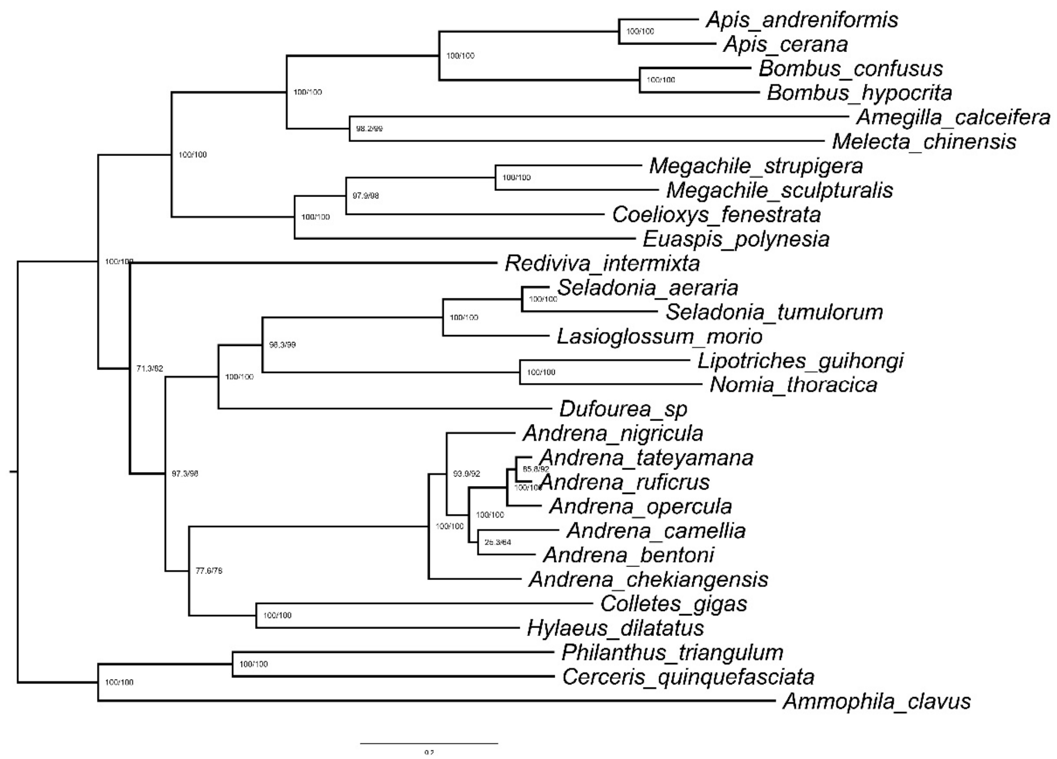

Figure S3. ML analysis of bees based on cds_faa using partitioned model in IQTREE. Nodes support values indicate SH-aLRT/UFBoot2.

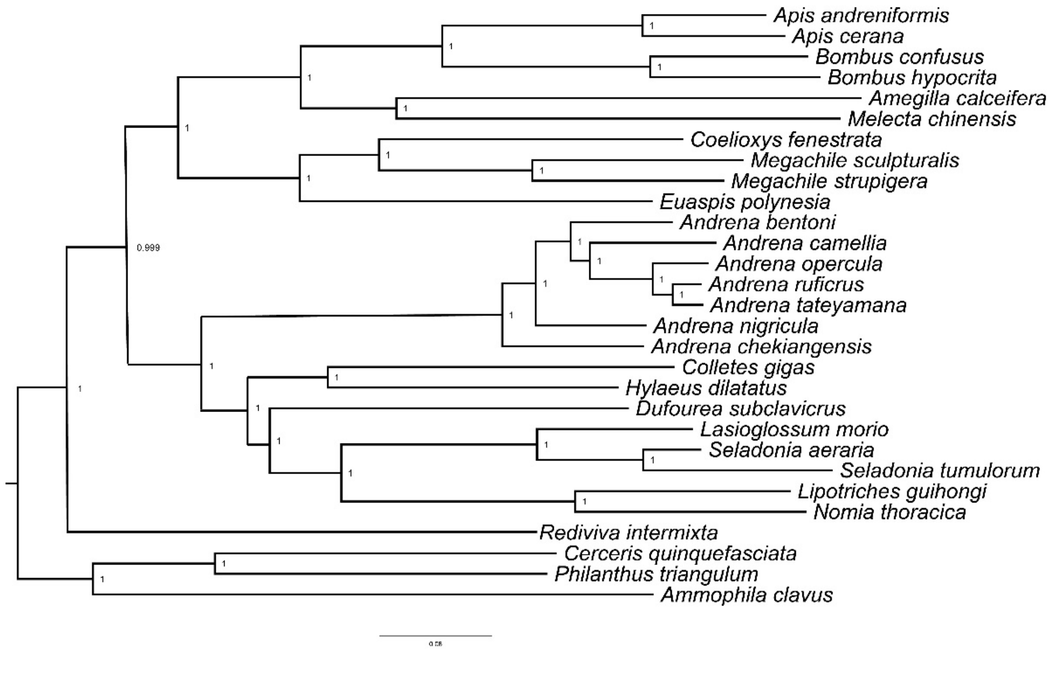

Figure S4. BI tree of bees based on cd_faa with a GTR + CAT model in phylobayes. Nodes support values indicate Bayesian posterior probabilities.

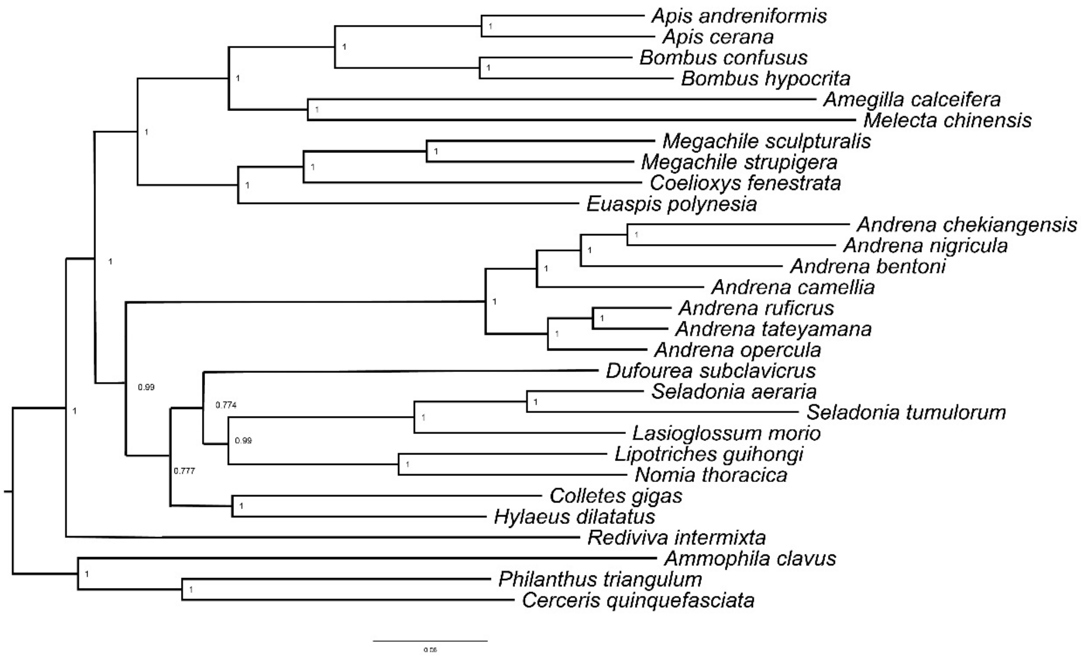

Figure S5. ML analysis of bees based on cds_rrna using partitioned model in IQTREE. Nodes support values indicate SH-aLRT/UFBoot2.

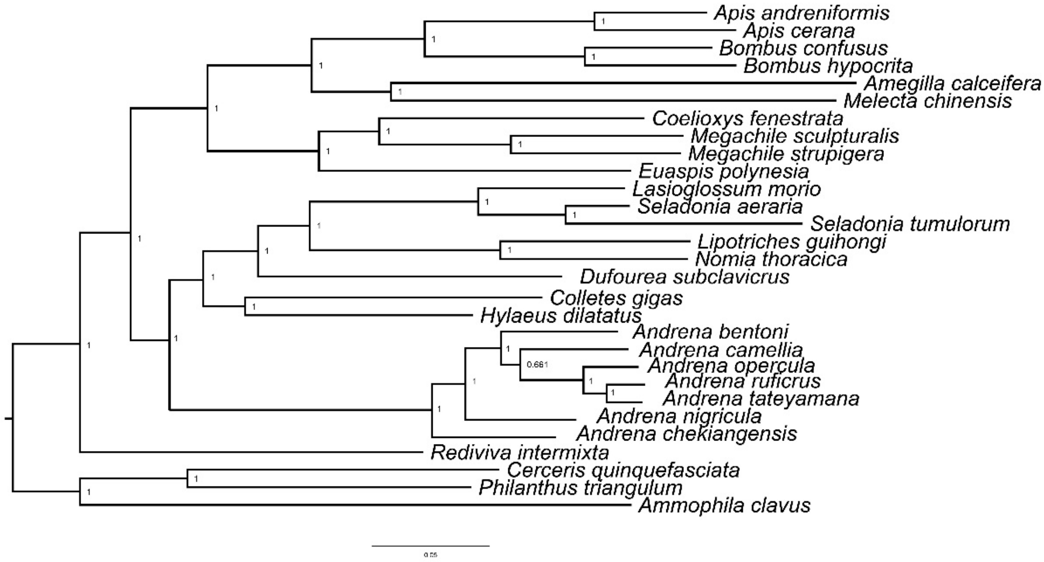

Figure S6. BI tree of bees based on cd_rrna with a GTR + CAT model in phylobayes. Nodes support values indicate Bayesian posterior probabilities.

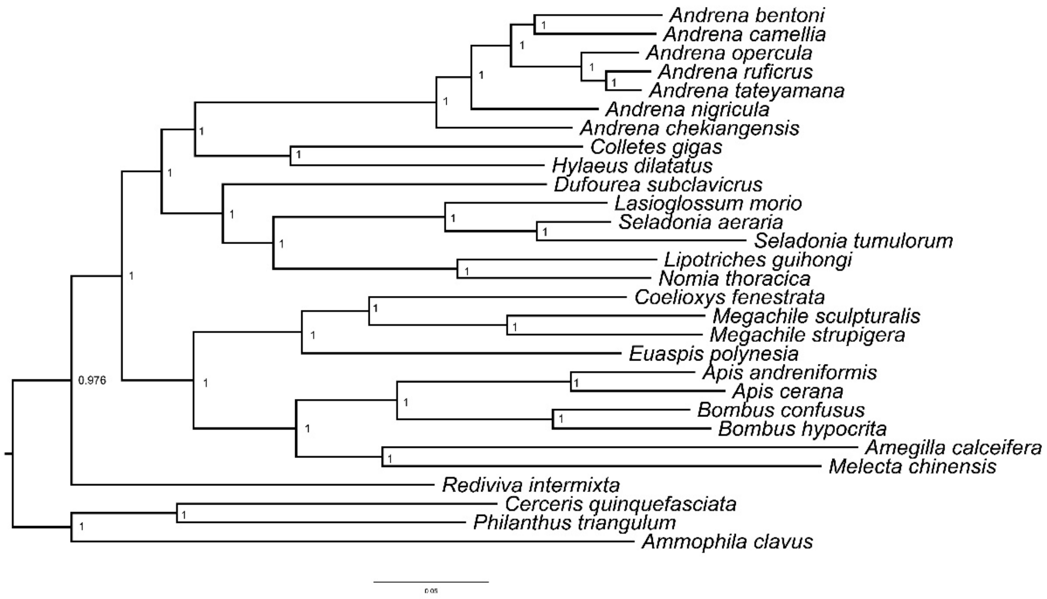

Figure S7. BI tree of bees based on cd_fna with a GTR + CAT model in phylobayes. Nodes support values indicate Bayesian posterior probabilities.

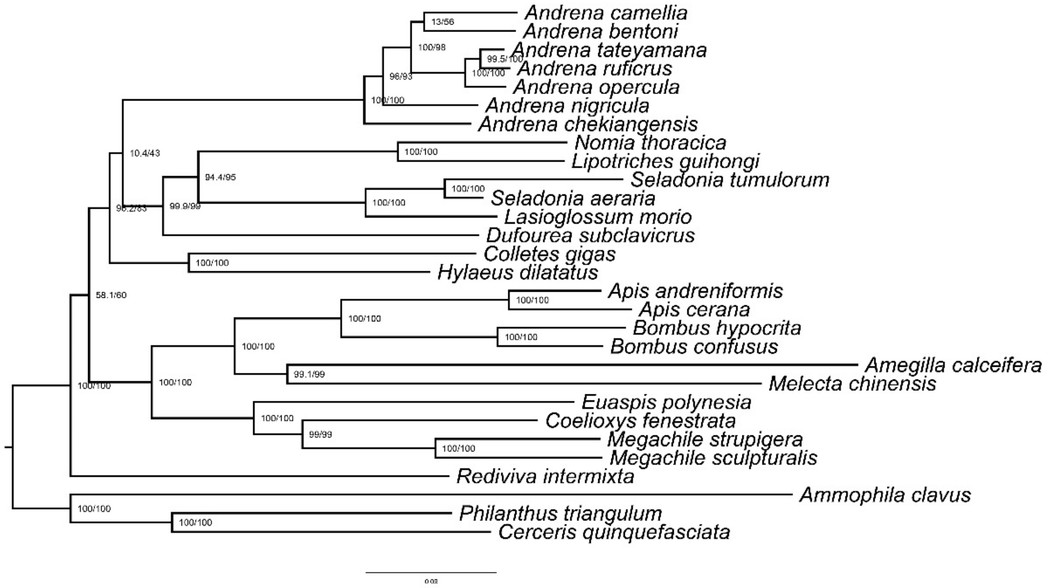

Figure S8. BI tree of bees based on cd12_fna with a GTR + CAT model in phylobayes. Nodes support values indicate Bayesian posterior probabilities.

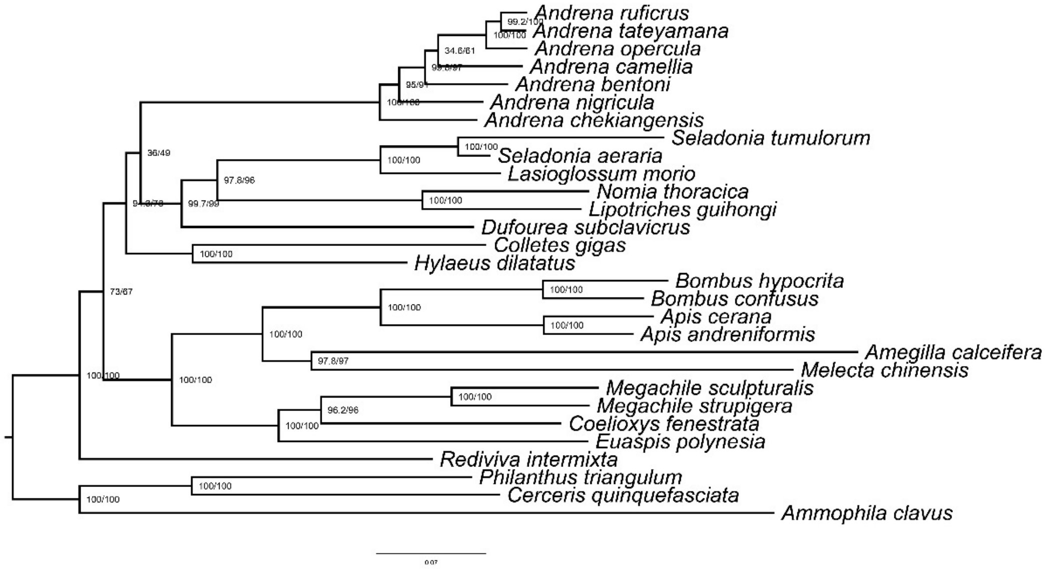

Figure S9. ML analysis of bees based on cds12_fna using partitioned model in IQTREE. Nodes support values indicate SH-aLRT/UFBoot2.

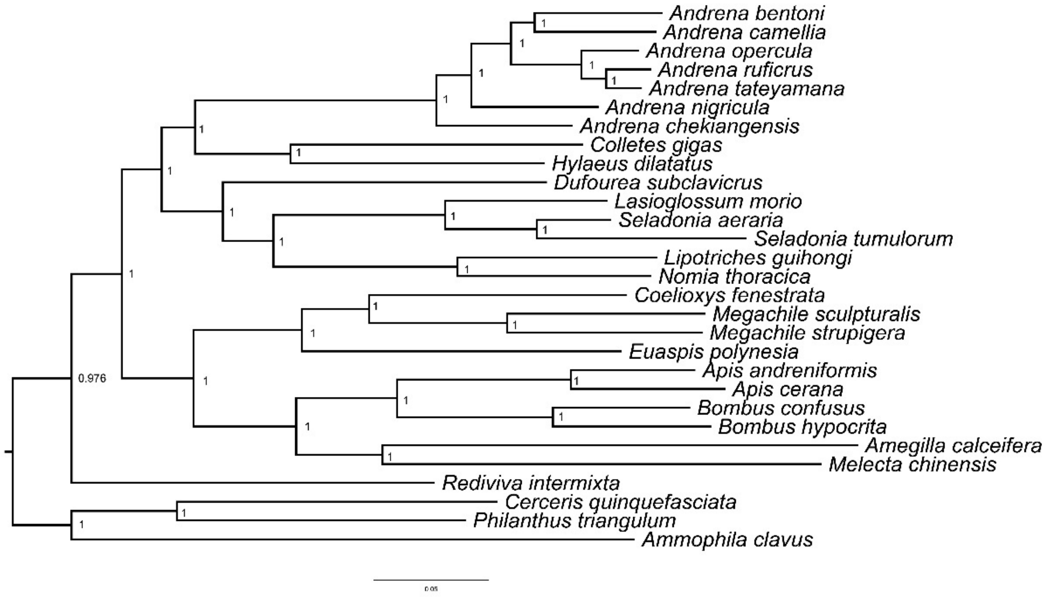

Figure S10. ML analysis of bees based on cds12_rrna using partitioned model in IQTREE. Nodes support values indicate SH-aLRT/UFBoot2.

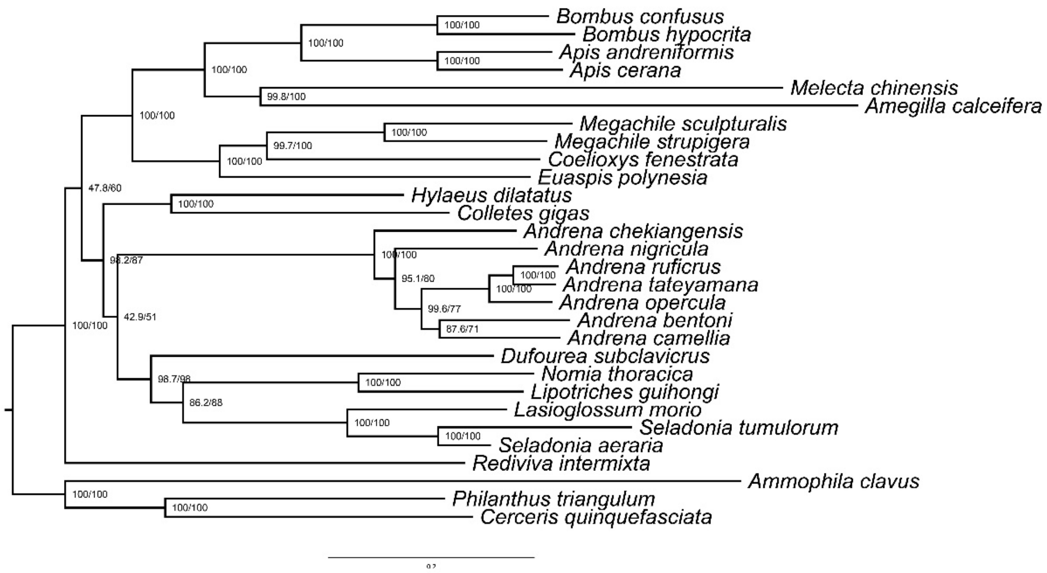

Figure S11. BI tree of bees based on cds_rrna with a GTR + CAT model in phylobayes. Nodes support values indicate Bayesian posterior probabilities.
